# Colorful Luminescence of Conjugated Polyelectrolytes Induced by Molecular Weight

**DOI:** 10.3390/polym14245372

**Published:** 2022-12-08

**Authors:** Kunsheng Wang, Yueqin Shi, Zhengjun Li

**Affiliations:** College of Materials & Environmental Engineering, Hangzhou Dianzi University, Xiasha Higher Education Zone, Hangzhou 310018, China

**Keywords:** aggregates, luminescence, conjugated polyelectrolytes, optoelectronic properties

## Abstract

Due to their distinctive intrinsic advantages, the nanoaggregates of conjugated polyelectrolytes (CPEs) are fascinating and attractive for various luminescence applications. Generally, the emission luminescence of CPEs is determined by the conjugated backbone structure, i.e., different conjugated backbones of CPEs produce emission luminescence with different emission wavelength bands. Here, we polymerized the bis(boronic ester) of benzothiadiazole and an alkyl sulfonate sodium-substituted dibromobenzothiatriazole to provide PBTBTz-SO_3_Na with different molecular weights via controlling the ratio of the monomer and the catalyst. Theoretically, the CPEs with the same molecular structure usually display similar photoelectronic performances. However, the resulting PBTBTz-SO_3_Na reveal a similar light absorption property, but different luminescence. The higher molecular weight is, the stronger the fluorescence intensity of PBTBTz-SO_3_Na that occurs. PBTBTz-SO_3_Na with different molecular weights have different colors of luminescence. It is well known that the molecular aggregates often led to weaker luminescent properties for most of the conjugated polymers. However, PBTBTz-SO_3_Na exhibits a higher molecular weight with an increasing molecular chain aggregation, i.e., the nanoaggregates of PBTBTz-SO_3_Na are beneficial to emission luminescence. This work provides a new possible chemical design of CPEs with a controllable, variable luminescence for further optoelectronics and biomedicine applications.

## 1. Introduction

Conjugated polyelectrolytes (CPEs) with a conjugated backbone and a charged flexible side-chain are employed because of their strong light-harvesting ability, excellent water solubility and good photo-stability, etc. [1,2,3,4,5]. The CPEs possess the properties of organic semiconductors, providing superior optoelectronic performances in virtue of the them having the same type of electronic delocalization based on the rigid conjugated backbones, which makes the utilization of CPEs possible in the fields of biomedicine and bioimaging, etc. [6,7,8,9,10,11,12]. The CPEs in polar media provide possible greener alternatives for the substitution of toxic solvents, i.e., their special solubilities allow for the possible fabrication of multilayer electronic devices in combination with the nonpolar conjugated polymers [4,5,13].

The powerful applications promote the design and synthesis of CPEs through the structural modifications of the side groups, the conjugated backbone, the ionic counterions and their functionalities [13,14,15,16,17,18,19]. The side groups permit one to modify the average distance between the charged pendants and the conjugated backbone. Charged pendants can be anionic or cationic groups, which have a structural handle to modulate the preferential interactions with the charged surfaces. Local electrostatic fields arising from the charged pendants result in the aggregates’ formation and charge transfer to take place among the inter- and intra- molecules [16,17,18,19]. The counterions that compensate for the charged pendants which are attached to the CPEs play an important role in regulating the charge carriers of the corresponding photovoltaic devices [19,20,21,22]. The conjugated backbone determines the primary optical properties of the CPEs [20,21,22]. Additionally, the conjugated length is also a non-ignorable element in the charge transfer among the molecular chains and the optical performance of the CPEs.

Developing innovative aggregated states are also another important consideration that aim to keep abreast with the development of the applied fields for CPEs [16]. The aggregate states have been suspected to be an important factor in determining the intra- and inter-molecular charge transfers of CPEs in the biological and optoelectronic devices [16,17]. Generally, the aggregate states of the CPEs are determined by the chemical structure of the conjugated backbones and the side groups [16,17,18]. There are few reports on the influence of the length of the conjugated backbone on their aggregation states, the molecular charge transfer and the optoelectronic performances of CPEs.

Benzotriazole (BTz) and benzothiadiazole (BT) have been proven to be two of the most promising units for the D-A conjugated polymers which are used in efficient optoelectronic devices because of their strong electron affinity [20,21,22,23]. E.g., the luminescence performances of BTz- and BT-containing copolymers can be regulated via the chemical structure change of the main and side chains, which is an important theoretical basis for optoelectronic applications [24,25,26,27,28,29]. BTz is an electron-deficient unit that can be modified via an alkyl side chain of the middle nitrogen atom on the triazole ring. The neutral homopolymer based on BTz backbones limits its solubility in common organic solvents, further restricting its application in the optoelectronic device, etc. The incorporating conjugate unit and ionic side chain onto the BTz backbone increase the solubilizing and planarity property of its copolymer [13,14]. In addition, these water-soluble polymers with variable shapes, sizes and functionalities can be further constructed by the self-assembly approach of the conjugated molecular long chain [4,5,6,7,8,9]. Their aggregate formation, twisted inter- and intra-molecular charge transfers and optoelectronic performance of the copolymers based on the BTz and BT backbone can be finely regulated without additional assist assembly [30,31,32,33,34,35,36].

Herein, we report CPEs (PBTBTz-SO_3_Na) with a sulfonate sodium charged-end side chain, but they are based on the BTz and BT conjugated backbones with different molecular weights. The resulting PBTBTz-SO_3_Na reveal similar light absorption properties, but they have different fluorescence performances. By measuring their optoelectronic properties along with the morphology characteristics, which were determined by the UV-vis absorption, photoluminescence (PL) and transmission electron microscopy (TEM), etc., we can further investigate the relationship among the lengths of the conjugate backbone, the molecular aggregated states and the luminescence performances of PBTBTz-SO_3_Na with various molecular weights. As a result, the molecular weight of PBTBTz-SO_3_Na plays a critical role in the intrachain and interchain energy transfer, which result from the various aggregates and electrostatic stabilization by the charges on the backbone. Such results will be provide the information about the structure–property relationship, and further insights into the working mechanisms will be gained for promising luminescence applications of CPEs.

## 2. Results and Discussions

**Scheme 1:**
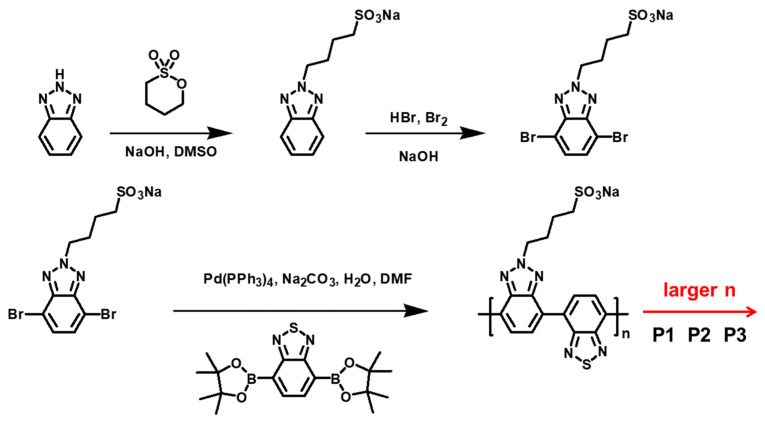
The synthesis of PBTBTz-SO_3_Na and the corresponding PBTBTz-SO_3_Na with larger molecular weights for P1, P2 and P3.

**Scheme 2:**
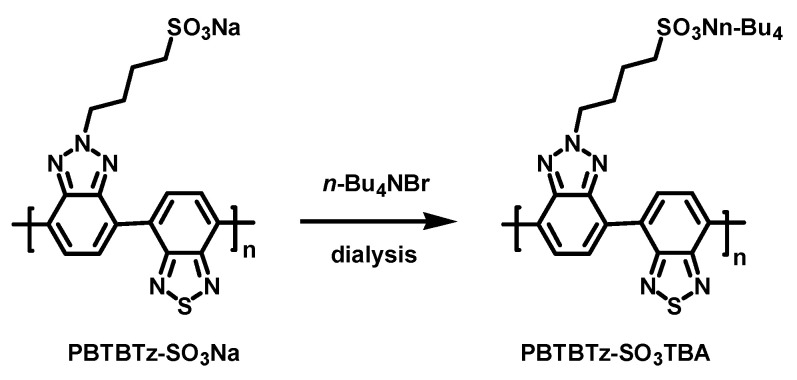
The synthesis of PBTBTz-SO_3_TBA.

The synthetic procedure and chemical structure of PBTBTz-SO_3_Na are exhibited in Scheme 1. Monomer BTzBr_2_-SO_3_Na was synthesized from the commercially available benzotriazole in two steps, including the alkylation and brominated reactions. Briefly, Suzuki coupling polymerization of the bis(boronic ester) of benzothiadiazole and an alkyl sulfonate sodium-substituted dibromobenzothiatriazole yielded PBTBTz-SO_3_Na, which is soluble in highly polar solvents, such as water and methanol, etc. All of the synthetic preparations and representations are demonstrated in detail in Figures S1–S4. The PBTBTz-SO_3_Na with different molecular weights was achieved by controlling the ratio of the monomer and the catalyst as shown in the SI. The ion exchange step further generated PBTBTz-SO_3_TBA through the exchange procedure in Scheme 2, in which the original sodium counter-cations were replaced with the tetrabutylammonium (TBA) to increase the solubility of the organic solvents, which was used for the following gel permeation chromatography (GPC) measurement in the DMF solvent [21,22].

**Figure 1:**
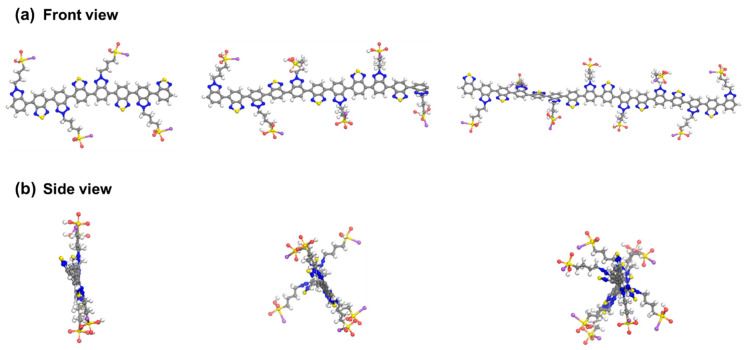
The front and side views of PBTBTz-SO_3_Na with the gradual increase of conjugated backbone length.

To examine the role of molecular weight, the ion-charged end and backbone structure of PBTBTz-SO_3_Na were maintained. The PBTBTz-SO_3_Na with larger molecular weights for P1, P2 and P3 were designed as per Scheme 1. The front and side views of PBTBTz-SO_3_Na with different lengths of conjugated backbone are exhibited in Figure 1. With the lengthening of the conjugated backbones, the molecular chains perform a highly ordered arrangement, and the twist of the molecular chains shows a gradual increase.

**Figure 2:**
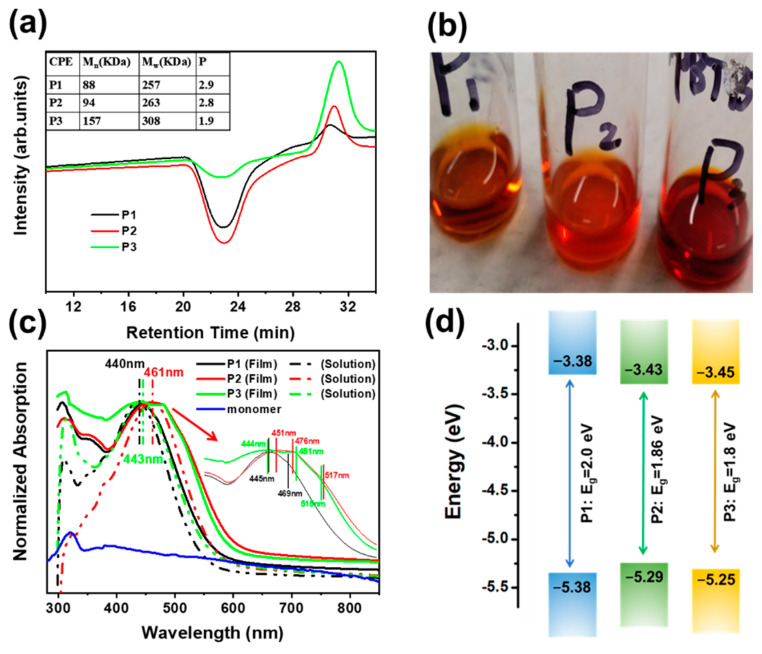
(**a**) The GPC of P1, P2 and P3. (**b**) The photograph of P1, P2 and P3 (5 mg/mL) solutions. (**c**) The normalized absorptions of P1, P2 and P3 in solution and thin film. The inset illustration shows the enlarged absorptions of P1, P2 and P3 films. (**d**) Energy level diagram of P1, P2 and P3.

The number average molecular weights of P1, P2 and P3 were obtained, and these have values of 88 kDa, 94 kDa and 157 kDa, respectively, by GPC in Figure 2a and Figures S5–S7. The GPC of all of the samples were measured using DMF as the solvent. We note that the aggregate of the polymer chains in the solution also influences the molecular weight. The photograph of the P1, P2 and P3 solutions is exhibited in Figure 2b. Figure 2c displays the optical absorption spectra of the P1, P2 and P3 solutions and thin films. The corresponding absorption peaks of the P1, P2 and P3 solutions and thin films are summarized in Table 1. The P1, P2 and P3 solutions display similar UV-vis absorption curves with maximal absorption peaks at 440 nm, 461 nm and 443 nm, respectively. However, as seen from the inset illustration, the thin film of P1 reveals a red-shifted absorption peak at 445 nm and a second absorption peak at 469 nm compared to the P1 solution, which are typically associated with the presence of aggregated chains. The maximal absorption peak at 445 nm corresponds to the intramolecular charge transfer band. The absorption spectra of the P2 thin film exhibits a blue-shifted absorption peak at 451 nm and another two red-shifted peaks at 476 nm and 517 nm, respectively, which is in contrast to the P2 solution, revealing that the aggregate of the P2 thin film increases with the lengthing of the conjugated backbone. P3 also shows three absorption peaks, indicating that more ordered microstructures of PBTBTz-SO_3_Na appeared as the molecular weight increased [37]. The optical bandgaps of P1, P2 and P3 are 2.0, 1.9 and 1.8 eV, which were calculated from the absorption edges of the thin films.

The electrochemical properties of P1, P2 and P3 were investigated by the cyclic voltammetry in Figure S8. The highest occupied molecular orbital (HOMO) and the lowest unoccupied molecular orbital (LUMO) levels of P1, P2 and were are extrapolated, and these are −5.3/−3.3 eV, −5.2/−3.3 eV and −5.2/−3.4 eV, respectively, which are basically consistent with the optical bandgap results [38,39,40,41,42]. The larger conjugation length of PBTBTz-SO_3_Na is more inclined to obtain a smaller bandgap. 

**Figure 3:**
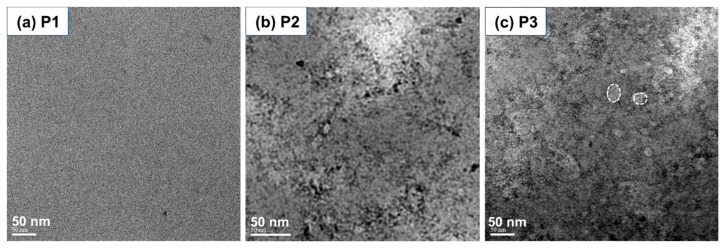
TEM images of (**a**) P1, (**b**) P2 and (**c**) P3.

The molecule chains aggregates of the P1, P2 and P3 films were performed to further study the relationship between the molecular weight and morphology by TEM [43,44,45,46]. As the molecular weight of PBTBTz-SO_3_Na increases, some small platelet-like molecular chains occur, as can be obviously seen in Figure 3 and Figure S9. The increasing platelet-like nanomorphology indicates the stronger aggregation of PBTBTz-SO_3_Na with a larger molecular weight, which is consistent with the trend represented by the GPC results.

**Figure 4:**
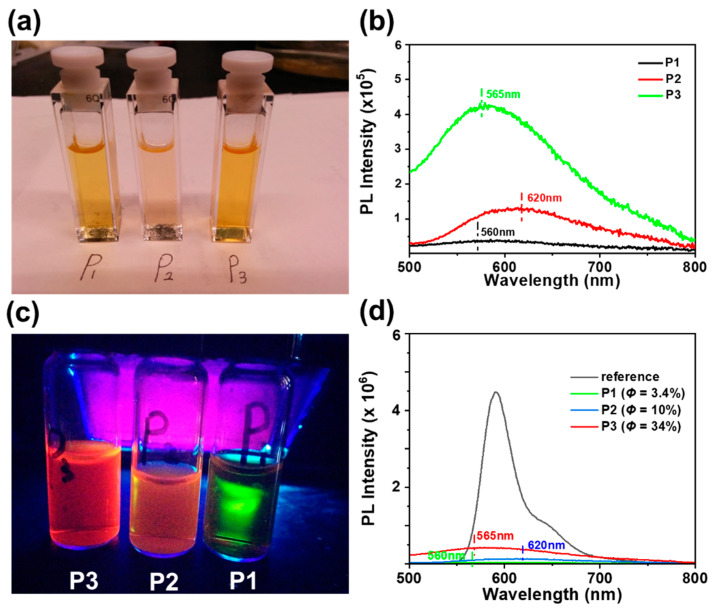
(**a**) The photograph of P1, P2 and P3 (0.016 mg/mL) solutions. (**b**) The PL intensity of P1, P2 and P3 in H_2_O with the concentration of 0.016 mg mL^−1^. (**c**) The fluorescence photograph of P1, P2 and P3 in H_2_O (5 mg/mL). (**d**) The integrated emissions of P1, P2 and P3 in H_2_O are used for the fluorescence quantum yield calculation with the concentration of 0.016 mg/mL.

The photoluminescence (PL) was further investigated to understand the relationship between the optical property and the molecular aggregated states of PBTBTz-SO_3_Na. The photograph of the P1, P2 and P3 (0.016 mg/mL) solutions used for the PL measurement is displayed in Figure 4a. P1, P2 and P3 reveal similar emissions under 430 nm irradiation in Figure 4b. It is worth mentioning that PBTBTz-SO_3_Na with a higher molecular weight exhibits a stronger fluorescence intensity, implying that the increasing molecular chain aggregation of PBTBTz-SO_3_Na is beneficial to the luminescence. As seen from the analysis results of λ_EX_, the higher the molecular weight is, the more ordered aggregations of PBTBTz-SO_3_Na occur. It is well known that interchain aggregation has led to the decreased fluorescence intensity for the most conjugated polymer [16,17,18,19,20]. The fluorescence spectra of the PBTBTz-SO_3_Na solution illustrates the opposite trend; the nanoaggregates result in a stronger luminescent emission. The emission maxima of P1, P2 and P3 are located at 560 nm, 620 nm and 565 nm, respectively. The fluorescence photograph of P1, P2 and P3 in H_2_O at concentrations of 5 mg/mL is shown in Figure 4c. PBTBTz-SO_3_Na with different molecular weights have different colors of luminescence. The fluorescence quantum yields of P1, P2 and P3 in H_2_O were determined relative to a reference sample with a known quantum yield. The reference sample here is rhodamine 101 inner salt in H_2_O. All of the PBTBTz-SO_3_Na solutions retains a strong fluorescence intensity, especially for the P2 and P3 solutions, and the fluorescence quantum yields of P1, P2 and P3 with the concentration of 0.016 mg/mL are 3.4%, 10% and 34%, respectively, as can be seen in Figure 4d. This phenomenon may be attributed to the twist of the molecular main chain, which inhibits the photoinduced electron transfer among the conjugated backbones.

## 3. Conclusions

PBTBTz-SO_3_Na samples with different molecular weights were achieved by controlling the polarity of the solvent and the ratio of the monomer and the catalyst. The molecular weight of PBTBTz-SO_3_Na plays a critical role in the intrachain and interchain energy transfer and photoluminescence as a result of the differences in the electrostatic stabilization by the charged pendants on the backbone. With the lengthening of the conjugated backbone, the molecular chain maintains a highly ordered arrangement, and the twist of the molecular main chain shows a gradual increase. The PBTBTz-SO_3_Na with a larger molecular weight shows more absorption peaks, indicating more ordered microstructures of PBTBTz-SO_3_Na have appeared as the molecular weight increases. Meanwhile, the increasing platelet-like nanomorphology indicates a stronger aggregation of PBTBTz-SO_3_Na with the larger molecular weight, as seen from the TEM mages. The PL is further investigated to understand the relationship between the optical property and molecular aggregated states of PBTBTz-SO_3_Na. PBTBTz-SO_3_Na with different molecular weights have different colors of luminescence. All of the PBTBTz-SO_3_Na solutions retain a strong fluorescence intensity, especially for the P2 and P3 solutions, and the fluorescence quantum yields of P1, P2 and P3 with the concentration of 0.016 mg/mL are 3.4%, 10% and 34%, respectively. The higher the molecular weight is, the stronger the aggregation and luminescence that can occur for PBTBTz-SO_3_Na. The phenomenon may be attributed to the twist of the molecular main chain, which inhibits the photoinduced electron transfer among the conjugated backbone.

## Figures and Tables

**Table 1:** The absorption peaks of P1, P2 and P3 solutions and thin films. The λ_EX_ is the abbreviation of corresponding absorption peak.

Polymers	Solution	Film		
	λ_EX2_ (nm)	λ_EX1_ (nm)	λ_EX2_ (nm)	λ_EX3_ (nm)
P1	440	445	469	\
P2	461	451	476	517
P3	443	444	481	515

## Data Availability

Not applicable.

## Supplementary Materials

The following supporting information can be downloaded at: https://www.mdpi.com/article/10.3390/polym14245372/s1, Figure S1: The ^1^H NMR spectrum of 2-(C_4_H_8_SO_3_Na)-2*H*-benzo[1,2,3]triazole in D_2_O; Figure S2: The mass spectrum of 2-(C_4_H_8_SO_3_Na)-2*H*-benzo[1,2,3]triazole in D_2_O; Figure S3: The ^1^H NMR spectrum of BTzBr_2_-SO_3_Na in D_2_O; Figure S4: The mass spectrum of BTzBr_2_-SO_3_Na in D_2_O; Figure S5: The original test picture of P1; Figure S6: The original test picture of P2; Figure S7: The original test picture of P3; Figure S8: The CV curves of P1, P2 and P3; Figure S9: The larger TEM images of P1, P2 and P3. Reference [47] are cited in the supplementary materials.

## References

[1] Johnston A.R., Minckler E.D., Shockley M.C.J., Matsushima L.N., Perry S.L., Ayzner A.L. (2022). Conjugated Polyelectrolyte-Based Complex Fluids as Aqueous Exciton Transport Networks. Angew. Chem. Int. Ed..

[2] Korley L.T.J., Epps T.H., Helms B.A., Ryan A.J. (2021). Toward polymer upcycling-adding value and tackling circularity. Science.

[3] Karst J., Floess M., Dingler M.C., Malacrida C., Steinle T., Ludwigs S., Hentschel M., Giessen H. (2021). Electrically switchable metallic polymer nanoantennas. Science.

[4] Chen Z., Huang Y., Gao J., Zhang L., Ma Z., Liu M., Emrick T., Liu Y. (2022). Synchronous Doping Effects if Cathode Interlayers on Efficient Organic Solar Cells. ACS Energy Lett..

[5] Jiang Y., Dong X., Sun L., Liu T., Qin F., Xie C., Jiang P., Hu L., Lu X., Zhou X. (2022). An alcohol-dispersed conducting polymer complex for fully printable organic solar cells with improved stability. Nat. Energy.

[6] Margossian K.O., Brown M.U., Emrick T., Muthukumar M. (2022). Coacervation in polyzwitterion-polyelectrolyte systems and their potential applications for gastrointestinal drug delivery platforms. Nat. Commun..

[7] Besford Q.A., Yong H., Merlitz H., Christofferson A.J., Sommer J.-U., Uhlmann P., Fery A. (2021). FRET-integrated polymer brushes for spatially resolved sensing of changes in polymer conformation. Angew. Chem. Int. Ed..

[8] Hlaváček A., Farka Z., Mickert M.J., Kostiv U., Brandmeier J.C., Horák D., Skládal P., Foret F., Gorris H.H. (2022). Bioconjugates of photon-upconversion nanoparticles for cancer biomarker detection and imaging. Nat. Protoc..

[9] Ishii Y., Matubayasi N., Watanabe G., Kato T., Washizu H. (2021). Molecular insights on confined water in the nanochannels of self-assembled ionic liquid crystal. Sci. Adv..

[10] Tsokkou D., Peterhans L., Cao D.X., Mai C.-K., Bazan G.C., Nguyen T.-Q., Banerji N. (2020). Excited State Dynamics of a Self-Doped Conjugated Polyelectrolyte. Adv. Funct. Mater..

[11] Friedowitz S., Lou J., Barker K.P., Will K., Xia Y., Qin J. (2021). Looping-in complexation and ion partitioning in nonstoichiometric polyelectrolyte mixtures. Sci. Adv..

[12] Xu Y., Li C., Lu S., Wang Z., Liu S., Yu X., Li X., Sun Y. (2022). Construction of emissive ruthenium (II) metallacycle over 1000 nm wavelength for in vivo biomedical applications. Nat. Commun..

[13] Au B.W.-C., Chan K.-Y. (2022). Towards an All-Solid-State Electrochromic Device: A Review of Solid-State Electrolytes and the Way Forward. Polymers.

[14] Armin A., Juska G., Philippa B.W., Burn P.L., Meredith P. (2013). Doping-induced screening of the built-in-field in organic solar cells: Effect on charge transport and recombination. Adv. Energy Mater..

[15] Bin H., Gao L., Zhang Z.G., Yang Y., Zhang Y. (2016). 11.4% efficiency non-fullerene polymer solar cells with trialkylsilyl substituted 2D-conjugated polymer as donor. Nat. Commun..

[16] Li Z., Jiang K., Yang G., Lai J.Y.L., Ma T. (2016). Donor polymer design enables efficient non-fullerene organic solar cells. Nat. Commun..

[17] Cui Q., Bazan G.C. (2018). Narrow band gap conjugated polyelectrolytes. Acc. Chem. Res..

[18] Osaka I., Zhang R., Sauvé G., Smilgies D.-M., Kowalewski T., McCullough R.D. (2015). High-lamellar Ordering and Amorphous-Like π-Network in Short-Chain Thiazolothiazole-Thiophene Copolymers Lead to High Mobilities. J. Am. Chem. Soc..

[19] Sohaimy M.I.H.M., Isa I.N. (2022). Proton-Conduction Biopolymer Electrolytes Based on Carboxymethyl Cellulose Doped with Ammonium Formate. Polymers.

[20] Baidurah S. (2022). Methods of Analyses for Biodegradable Polymers: A Review. Polymers.

[21] Shi Y., Mai C.K., Fronk S.L., Chen Y., Bazan G.C. (2016). Optical properties of benzotriazole-based conjugated polyelectrolytes. Macromolecules.

[22] Wilson J.S., Frampton M.J., Michels J.J., Sardone L., Marletta G., Friend R.H., Samorì P., Anderson H.L., Cacialli F. (2005). Supramolecular Complexes of Conjugated Polyelectrolytes with Poly(ethylene oxide): Multifunctional Luminescent Semiconductors Exhibiting Electronic and Ionic Transport. Adv. Mater..

[23] Shi Y., Tan L., Chen Y. (2014). Dye-sensitized nanoarrays with discotic liquid crystals as interlayer for high-efficiency inverted polymer solar cells. ACS Appl. Mater. Interface.

[24] Fronk S.L., Shi Y., Siefrid M., Mai C.K., McDowell C., Bazan G.C. (2016). Chiroptical properties of a benzotriazole-thiophene copolymer bearing chiral ethylhexyl side chains. Macromolecules.

[25] Peet J., Cho N.S., Lee S.K., Bazan G.C. (2008). Transition from solution to the solid state in polymer solar cells cast from mixed solvents. Macromolecules.

[26] Alaswad S.O., Mahmoud A.S., Arunachalam P. (2022). Recent Advances in Biodegradable Polymers and their Biological Applications: A Brief Review. Polymers.

[27] Chen X., Qin H., Qian X., Zhu W., Li B., Zhang B., Lu W., Li R., Zhang S., Zhu L. (2022). Relaxor ferroelectric polymer exhibits ultrahigh electromechanical coupling at low electric field. Science.

[28] Wade J., Hilfiker J.N., Brandt J.R., Liirò-Peluso L., Wan L., Shi X., Salerno F., Ryan S.T.J., Schöche S., Arteaga O. (2020). Natural optical activity as the origin of the large chiroptical properties in π-conjugated polymer thin films. Nat. Commun..

[29] Fu H., Yao J., Zhang M., Xue L., Zhou Q., Li S., Lei M., Meng L., Zhang Z.-G., Li Y. (2022). Low-cost synthesis of small molecule acceptors makes polymer solar cells commercially viable. Nat. Commun..

[30] Qi F., Jiang K., Lin F., Wu Z., Zhang H., Gao W., Li Y., Cai Z., Woo H.Y., Zhu Z. (2021). Over 17% Efficiency Binary Organic Solar Cells with Photoresponses Reaching 1000 nm Enabled by Selenophene-Fused Nonfullerene Acceptors. ACS Energy Lett..

[31] Feng C., Wang X., He Z., Cao Y. (2021). Formation Mechanism of PFN Dipole Interlayer in Organic Solar Cells. Sol. RRL.

[32] Ma Q., Jia Z., Meng L., Zhang J., Zhang H., Huang W., Yuan J., Gao F., Wan Y., Zhang Z. (2020). Promoting charge separation resulting in ternary organic solar cells efficiency over 17.5%. Nano Energy.

[33] Tybrandt K., Zozoulenko I.V., Berggren M. (2017). Chemical potential-electric double layer coupling in conjugated polymer-polyelectrolyte blends. Sci. Adv..

[34] Guo S., Garaj S., Bianco A., Ménard-Moyon C. (2022). Controlling covalent chemistry on graphene oxide. Nat. Rev. Phys..

[35] Raynold A.A.M., Li D., Chang L., Gautrot J.E. (2021). Competitive binding and molecular crowding regulate the cytoplasmic interactome of non-viral polymeric gene delivery vectors. Nat. Commun..

[36] Liao P., Zang S., Wu T., Jin H., Wang W., Huang J., Tang B.Z., Yan Y. (2021). Generating circularly polarized luminescence from clusterization-triggerd emission using solid phase molecular self-assembly. Nat. Commun..

[37] Wan Y., Ramirez F., Zhang X., Nguyen T.-Q., Bazan G.C., Lu G. (2021). Data driven discovery of conjugated polyelectrolytes for optoelectronic and photocatalytic applications. NPJ Comput. Mater..

[38] Tan C., Pinto M.R., Kose M.E., Ghiviriga I., Schanze K.S. (2004). Solvent-induced self-assembly of a meta-linked conjugated polyelectrolyte. Helix formation, guest intercalation and amplified quenching. Adv. Mater..

[39] Chambers J.E., Zubkov N., KubÁnkovÁ M., Nixon-Abell J., Mela I., Abreu S., Schwiening M., Lavarda G., López-Duarte I., Kuimova M.K. (2022). Z-α1-antitrypsin polymers impose molecular filtration in the endoplasmic reticulum after undergoing phase transition to a solid state. Sci. Adv..

[40] Cha H., Wheeler S., Holliday S., Dimitrov S.D., Wadsworth A., Lee H.H., Baran D., McCulloch I., Durrant J.R. (2018). Influence of blend morphology and energetics on charge separation and recombination dynamics in organic solar cells incorporating a nonfullerene acceptor. Adv. Funct. Mater..

[41] Lahiri S., Thompson J.L., Moore J.S. (2000). Solvophobically driven π-stacking of phenylene ethynylene macrocycles and oligomers. J. Am. Chem. Soc..

[42] Nguyen-Dang T., Chae S., Chatsirisupachai J., Wakidi H., Promarak V., Visell Y., Nguyen T.-Q. (2022). Dual-mode organic electrochemical transistors based on self-doped conjugated polyelectrolytes for reconfigurable electronics. Adv. Mater..

[43] Li T., Feng C., Yap B.K., Zhu X., Xiong B., He Z., Wong W.-Y. (2021). Accelerating charge transfer via nonconjugated polyelectrolyte interlayers toward efficient versatile photoredox catalysis. Commun. Chem..

[44] Koh Q.-M., Tang C.G., Ang M.C.-Y., Choo K.-K., Seah Q.-J., Png R.-Q., Chua L.-L., Ho P.K.H. (2021). Overcoming the water oxidative limit for ultra-high-workfunction hole-doped polymers. Nat. Commun..

[45] Wang Z.-X., Liao W.-Q. (2022). Giant electromechanical effects in polymers. Science.

[46] Jia M., Zhang J. (2022). Thermoresponsive PEDOT:PSS/PNIPAM conductive hydrogels as wearable resistive sensors for breathing pattern detection. Poly. J..

[47] Morrls J.V., Mahaney M.A., Huber J.R. (1976). Fluorescence Quantum Yield Determinations. 9,10-Diphenylanthracene as A Reference Standard in Different Solvents. J. Phys. Chem..

