# Supplementary material for: Colorful Luminescence of Conjugated Polyelectrolytes Induced by Molecular Weight
Source: Polymers (Basel). 2022 Dec 8;14(24):5372. doi: 10.3390/polym14245372 (PMC9785289; doi:10.3390/polym14245372)
Supplement: Supplementary file 1 [file polymers-14-05372-s001.zip › polymers-2023251-supplementary.pdf]

polymers

Supporting Information

Colorful Luminescence of Conjugated Polyelectrolytes Induced by Molecular Weight

Kunsheng Wang †, Yueqin Shi *,† and Zhengjun Li

College of Materials & Environmental Engineering, Hangzhou Dianzi University, Xiasha Higher Education Zone, Hangzhou 310018, China
* Correspondence: yshi@hdu.edu.cn
† Kunsheng Wang and Yueqin Shi contributed equally to this work.

Synthesis of 2-($C_4H_8SO_3Na$)-2*H*-benzo[1,2,3]triazole

Benzotriazole (28.6 mmol, 3.4 g) and tetrabutylammonium bromide (460 mg, 1.43 mmol) were dissolved in 140 mL anhydrous DMSO, which was degassed by N_2 for about 5 min. A 16 mL NaOH (14 g) aqueous solution was added using a syringe, which was followed by the addition of 1,4-butanesultone (3500 μL, 66.8 mmol). After stirring for 3 hours at room temperature, the reaction mixture was poured into acetone. After filtration and washing it with acetone, the white precipitate was obtained. The purification by chro-matography (reversed phase silica gel C-18, 10:1 H_2O:MeOH) provided the title product as a white solid (41% yield). ^{1}H NMR (500 MHz, D_2O) δ 7.53 (d, 2H), 7.86 (d, 2H), 4.79 (t, 2H), 2.89 (t, 2H), 2.24 (m, 2H), 1.74 (m, 2H). MS m/z calcd for $C_{10}H_{12}N_3SO_3Na$: 277 g/mol found: 254.29 ($C_{10}H_{12}N_3SO_3^-$). See below spectra in Figures S1 and S2, which were cited from [1].

Synthesis of 4,7-dibromo-2-($C_4H_8SO_3Na$)-2*H*-benzo[1,2,3]triazole ($BTzBr_2$-SO_3Na)

BTz-$C_4H_8SO_3Na$ (0.73 mmol, 0.201 g) and an aqueous HBr solution (33 wt%, 1.3 mL) were added to a round bottom flask, and the mixture was stirred for one hour at 100 °C. After cooling it down to room temperature, bromine (0.33 g, 0.106 mL) was added. The solution was stirred for 12 h at 80 °C. After cooling it down to room temperature again, an aqueous solution of $NaHCO_3$ was added, and the reaction was stirred in another 2 hours. The purification by chromatography (reversed phase silica gel C-18, 10:1 H_2O:MeOH) provided the title product as a white solid (83% yield). ^{1}H NMR (400 MHz, D_2O) δ 7.59 (s, 2H), 4.88 (t, 2H), 2.90 (t, 2H), 2.25 (m, 2H), 1.74 (m, 2H). MS m/z calcd for $C_{10}H_{10}Br_2N_3SO_3Na$: 435 g/mol found: 411.71 ($C_{10}H_{10}Br_2N_3SO_3^-$). See below spectra in Figures S3 and S4, which were cited from [1].

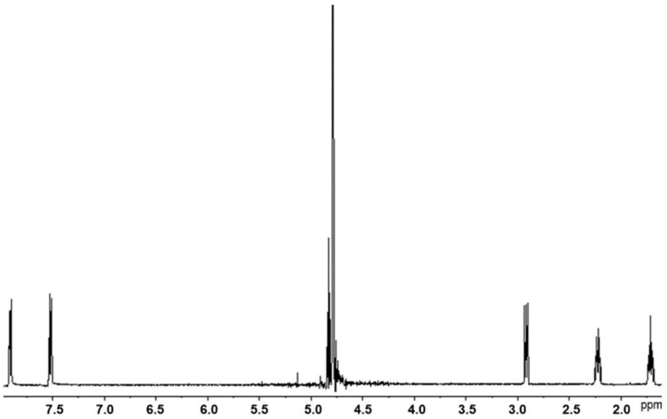

Figure S1. The ^{1}H NMR spectrum of 2-($C_4H_8SO_3Na$)-2*H*-benzo[1,2,3]triazole in D_2O.

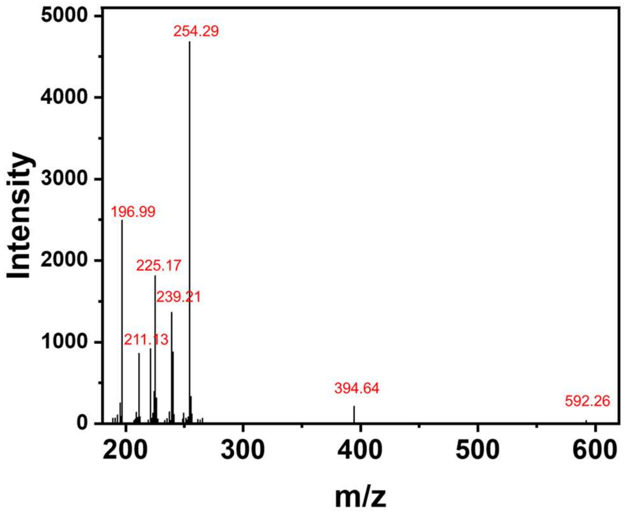

Figure S2. The mass spectrum of 2-($C_4H_8SO_3Na$)-2*H*-benzo[1,2,3]triazole in D_2O.

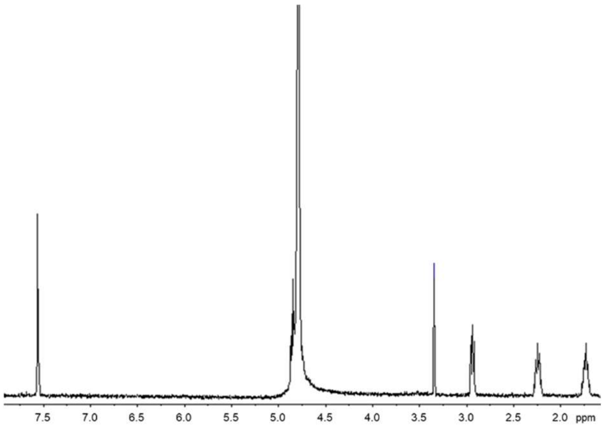

Figure S3. The ^1H NMR spectrum of $BTzBr_2$-SO_3Na in D_2O.

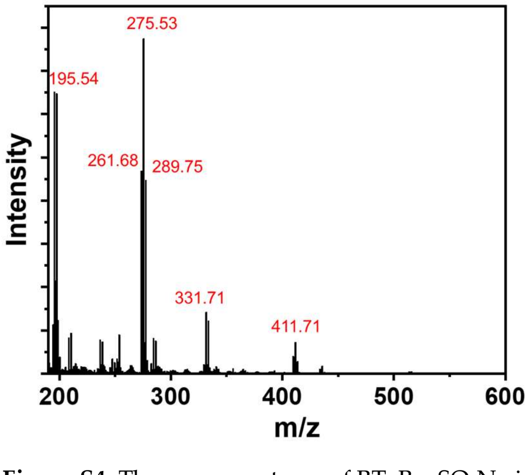

Figure S4. The mass spectrum of $BTzBr_2$-SO_3Na in D_2O.

Suzuki coupling polymerization of PBTBTz-SO_3Na with different molecular weights

Dibromide (1.0 equiv), diboronic ester (1.0 equiv), Na_2CO_3 (5 equiv), and $Pd(PPh_3)_4$ (2 mol%) were combined in the microwave tube using a stir bar in a glove box. The tube was sealed and taken out of the glove box. A mixture of DMF and H_2O (4/1 vol/vol) was degassed with argon for 5 min, then, it was transferred into the above tube under an argon atmosphere. The solution was placed into an oil bath, and heated to 80 ºC, at which temperature the mixture turned dark red in ~20 min. The reaction was conducted at about 80

ºC for 24 h, and it was poured into acetone. The red precipitate was collected by filtration and washed with acetone and MeOH. The red products were dissolved in millipore H_2O, and then, they were transferred into a dialysis tube (MWCO: 3500-5000). The tube was placed into the millipore H_2O for dialysis which took place after a week, and the millipore H_2O was changed every 12 h. After freeze-drying, the P1 was obtained as a dark red solid.

Dibromide (1.0 equiv), diboronic ester (1.0 equiv), Na_2CO_3 (5 equiv), and $Pd(PPh_3)_4$ (1.8 mol%) were combined in the microwave tube using a stir bar in a glove box. The tube was sealed and taken out of the glove box. A mixture of DMF and H_2O (3/1 vol/vol) was degassed with argon for 5 min, and then, it was transferred into the above tube under an argon atmosphere. The solution was placed into an oil bath and heated to 80 ºC, at which temperature the mixture turned dark red in ~20 min. The reaction was conducted at about 80 ºC for 24 h, and it was poured into acetone. The red precipitate was collected by filtration and washed with acetone and MeOH. The red products were dissolved in millipore H_2O, and then, they were transferred into a dialysis tube (MWCO: 3500-5000). The tube was placed into the millipore H_2O for dialysis which took place after a week, and the millipore H_2O was changed every 12 h. After freeze-drying, the P2 was obtained as a dark red solid.

Dibromide (1.0 equiv), diboronic ester (1.0 equiv), Na_2CO_3 (5 equiv), and $Pd(PPh_3)_4$ (1.5 mol%) were combined in the microwave tube using a stir bar in a glove box. The tube was sealed and taken out of the glove box. A mixture of DMF and H_2O (3/1 vol/vol) was degassed with argon for 5 min, and then, it was transferred into the above tube under an argon atmosphere. The solution was placed into an oil bath and heated to 80 ºC, at which temperature the mixture turned dark red in ~20 min. The reaction was conducted at about 80 ºC for 24 h, and it was poured into acetone. The red precipitate was collected by filtration and washed with acetone and MeOH. The red products were dissolved in millipore H_2O, and then, they were transferred into a dialysis tube (MWCO: 3500-5000). The tube was placed into the millipore H_2O for dialysis which took place after a week, and the millipore H_2O was changed every 12 h. After freeze-drying, the P3 was obtained as a dark red solid.

Characterizations

General Information for Synthesis

All of the glasses were oven dried. The chemicals were commercially available, and they were used as received. Thin layer chromatography (TLC) was performed using EM Science Silica Gel 60 F254 glass plates. Flash chromatography was performed using 60 Å silica gel (37-75 μm). The dialysis membrane (MWCO: 3500-5000 Da) was purchased from Spectrum® Laboratories Inc. The ^{1}H NMR spectra were recorded at either 400 MHz or 500 MHz in D_2O. The GPC of PBTBTz-SO_3TBA was measured using DMF as the solvent.

Cyclic voltammetry (CV) measurements

The cyclic voltammetry (CV) was performed using a CHI instrument model 730B in a standard three-electrode, with one compartment configuration being equipped with a Ag wire quasi reference electrode, a Pt wire counter electrode, and a glassy carbon working electrode. Additionally, the glass carbon electrodes were polished with alumina. The polyelectrolytes formed the films by dripping 5 μL of 5 mg/mL solution on the carbon working electrode, then, they were dried under a vacuum overnight before we took the measurements. The CV experiments were processed in anhydrous acetonitrile solution (PBTBTz-SO_3Na was performed in DMF) with 0.1 M tetrabutylammonium hexafluorophosphate ($TBAPF_6$) as the supporting electrolyte at a scan rate of 100 mV/s. The electrochemical solutions were purged with argon for 15 minutes to deoxygenate the solutions. Ferrocene was used as the internal standard.

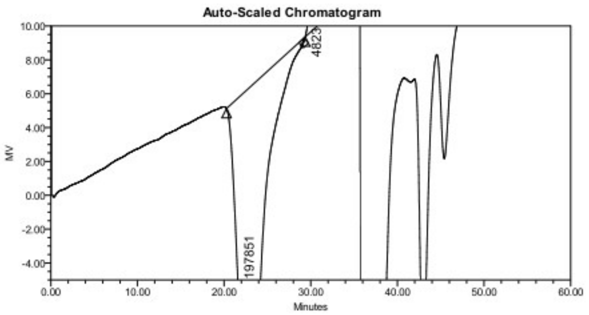

GPC Results

	Dist Name	Mn	Mw	MP	Mz	Mz+1	Mv	Polydispersity	MW Marker 1	MW Marker 2
1		88362	257047	197851	576003	964626		2.909027		
2		4504	4728	4823	4948	5160		1.049658		
3										

Figure S5. The original test picture of P1.

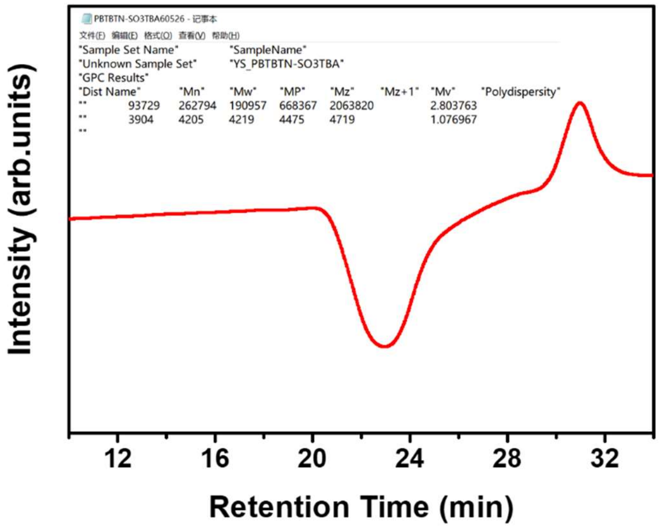

Figure S6. The original test picture of P2.

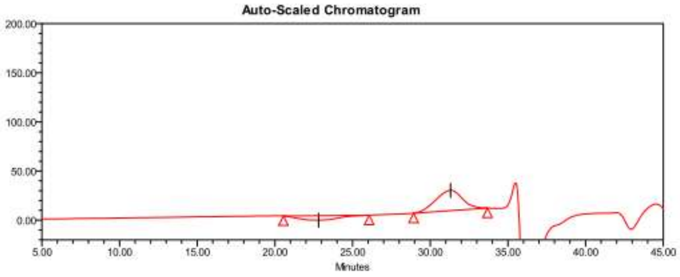

GPC Results

	Dist Name	Mn	Mw	MP	Mz	Mz+1	Mv	Polydispersity	MW Marker 1	MW Marker 2
1		157401	308505	215617	568810	854154		1.959990		
2		3572	4000	3679	4424	4838		1.119679		
3										

Processed Channel:

	Peak Name	Retention Time (min)	Area	% Area	Height
1	Peak3	22.814	787272	24.03	-4595
2	Peak4	31.315	2488513	75.97	20663
3	Peak2	36.476			

Figure S7. The original test picture of P3.

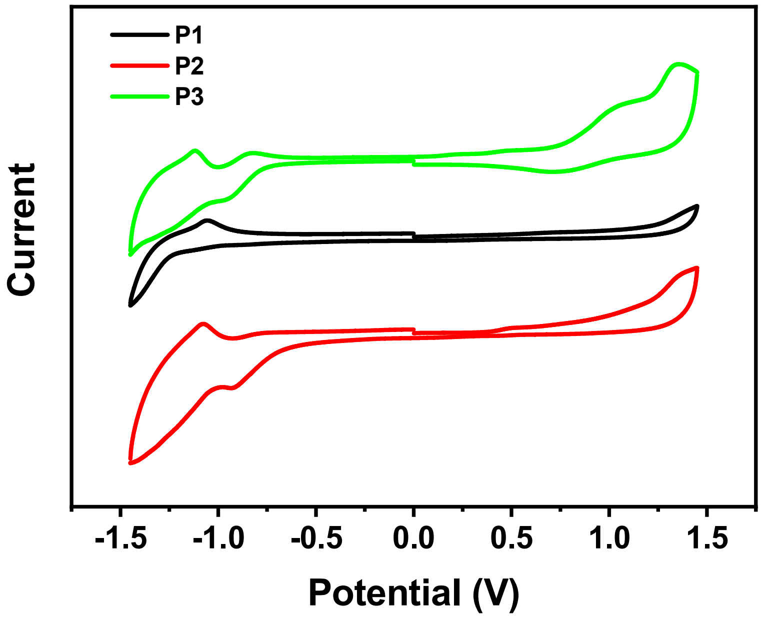

Figure S8. The CV curves of P1, P2 and P3.

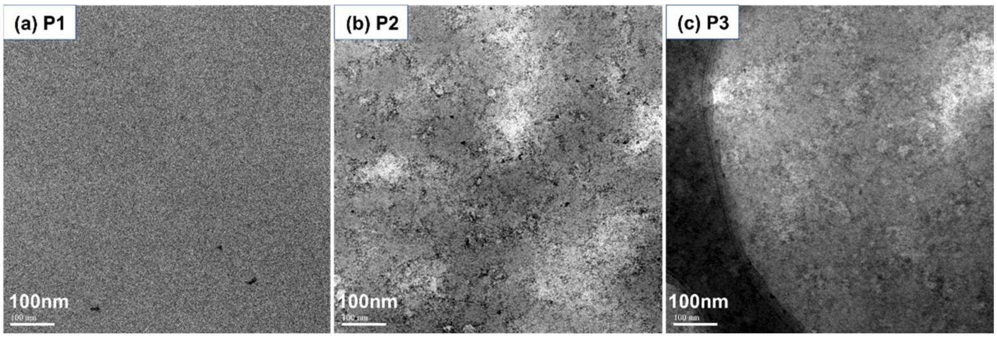

Figure S9. The larger TEM images of P1, P2 and P3.

UV-vis absorptions

The UV-vis absorption measurements were performed using a Perkin Elmer Lambda 750 spectrophotometer. The films were prepared by spin coating from the corresponding solutions (5 mg/mL) onto the glass substrates. The concentration of the solution was 0.016 mg/mL.

Fluorescence Quantum Yield (*Φ*)

The concentration of each sample was 0.016 mg mL^{-1}. The fluorescence quantum yields of P1, P2 and P3 in H_2O were determined relative to a reference sample of a known quantum yield ***Φ*** (rhodamine 101 inner salt in H_2O) with the possibility of correcting for the differences between the refractive index of the reference n_r and the sample solutions n_s using the following equation [2].

$$\Phi_s = \frac{S_s}{S_r}\frac{n_s^2}{n_r^2}\Phi_r$$

The subscripts s and r denote the samples and reference, respectively. The S represents the integral of the corrected emission spectra at the wavelength of excitation. The

n stands for the refractive index of the solution. The Φ is the fluorescence quantum yields. The Φ_r value of rhodamine 101 inner salt is 100%.

The integrated emission S was obtained in three steps. The first step was the UV-vis absorption of the sample and reference to obtain the excitation wavelength. Here, the excitation wavelengths were 440 nm, 461 nm and 443 nm for P1, P2 and P3, respectively. The second step was the fluorescence emission spectra of the sample, reference and blank. The third step was subtracting the blank from the sample and the reference, respectively, to obtain the fluorescence integrals of the sample and the reference shown in the Figure 4d.

References

1. Shi, Y.; Mai, C.-K.; Fronk, S.L.; Chen, Y.; Bazan, G.C. Optical properties of Benzotriazole-based Conjugated Polyelectrolytes. *Macromolecules* **2016**, *49*, 6343–6349.
2. Morrls, J.V.; Mahaney, M.A.; Huber, J.R. Fluorescence Quantum Yield Determinations. 9,10-Diphenylanthracene as A Reference Standard in Different Solvents. *J. Phys. Chem.* **1976**, *80*, 969–974.
